# Palliative care in the emergency department as seen by providers and users: a qualitative study

**DOI:** 10.1186/s13049-019-0662-y

**Published:** 2019-09-18

**Authors:** Silvia Di Leo, Sara Alquati, Cristina Autelitano, Massimo Costantini, Gianfranco Martucci, Francesco De Vincenzo, Beata Kuczynska, Alessandra Morini, Laura Trabucco, Raffaella Ursicelli, Gianluca Catania, Luca Ghirotto

**Affiliations:** 1 Psycho-oncology Unit, Azienda USL - IRCCS di Reggio Emilia, Reggio Emilia, Italy; 2 Palliative Care Unit, Azienda USL - IRCCS di Reggio Emilia, Reggio Emilia, Italy; 3 Scientific Directorate, Azienda USL - IRCCS di Reggio Emilia, Reggio Emilia, Italy; 4 Department of Emergency Medicine, Azienda USL - IRCCS di Reggio Emilia, Reggio Emilia, Italy; 5 Day Surgery, Azienda USL - IRCCS di Reggio Emilia, Reggio Emilia, Italy; 6 Department of Health Sciences, University of Genoa, Genova, Italy; 7 Qualitative Research Unit, Azienda USL - IRCCS di Reggio Emilia, Reggio Emilia, Italy

**Keywords:** Emergency Department, Palliative Care, Qualitative research, Healthcare professional, Patient, Relative, Improvement program

## Abstract

**Background:**

Much effort has been made to explore how patients with advanced chronic illness and their families experience care when they attend the Emergency Department, and many studies have investigated how healthcare professionals perceive Palliative Care provision in the Emergency Department. Various models exist, but nonetheless incorporating palliative care into the Emergency Department remains challenging. Considering both healthcare professionals’ and users’ perspective on problems encountered in delivering and receiving appropriate palliative care within this context may provide important insight into meaningful targets for improvements in quality of care. Accordingly, this study aims at exploring issues in delivering palliative care in the Emergency Department from the perspective of both providers and users, as part of a larger project on the development and implementation of a quality improvement program in Italian Emergency Departments.

**Methods:**

A qualitative study involving focus group interviews with Emergency Department professionals and semi-structured interviews with patients with palliative care needs in the Emergency Department and their relatives was conducted. Both datasets were analyzed using Thematic Analysis.

**Results:**

Twenty-one healthcare professionals, 6 patients and 5 relatives participated in this study. Five themes were identified: 1) shared priorities in Emergency Department among healthcare professionals and patients, 2) the information provided by healthcare professionals and that desired by relatives, 3) perception of environment and time, 4) limitations and barriers to the continuity of care, and 5) the contrasting interpretations of giving and receiving palliative care.

**Conclusions:**

This study provides insights into targets for changes in Italian Emergency Departments. Room for improvement relates to training for healthcare professionals on palliative care, the development of a shared care pathway for patients with palliative care needs, and the optimization of Emergency Department environment. These targets will be the basis for the development of a quality improvement program in Italian Emergency Departments.

## Introduction

Palliative Care (PC) is defined as the holistic care approach aimed at guaranteeing relief from suffering and improving quality of life for people with life-limiting illness, as well as their families [1]. In the last decade PC has been actively endorsed by the WHO, stressing both the medical and ethical aspects of maintaining best quality of life possible, while responding to unmet needs in chronic pain and end-of-life care. In general, PC is provided within oncology units, but it has increasingly expanded its operational scope to Emergency Departments (EDs) [2–12]. Although many ED healthcare professionals (HPs) recognize the importance of PC provision in the ED [13–16], and various model exist on incorporating PC into the ED [17–19], some conceive PC to be beyond of their scope or even at odds with the principles of emergency medicine [13, 16, 20–24]. This has been documented in two specific surveys [22, 25], whose results evidence that PC is eventually provided to a limited number of patients presenting to the ED, presumably much fewer than those actually in need. Criticalities of PC in the ED setting have also been reported by other studies in literature considering the users’ perspective, documenting the patients’ dissatisfaction with PC in the ED. [4, 6, 26, 27]

Considering both professionals’ and users’ perspective on problems encountered in delivering and receiving appropriate PC within the ED in the context of a chronic advanced illness may provide important insight into meaningful targets for improvements in quality of care.

In the attempt to identify the critical issues in PC implementation in the ED and work towards the improvement in quality of care provided, we decided to explore the matter from the perspective of both providers (the ED professionals) and end-users (i.e. patients with chronic advanced illness visiting the ED and their relatives). The study presented herein is part of a larger project addressing the development and implementation of a PC quality improvement program in Italian EDs, according to the MRC Framework for the development of complex interventions [28].

## Methods

### Study design and setting

This was a generic qualitative research [29] we conducted in the ED of our institution, a 900-bed public research Hospital in Northern Italy, recently awarded the title of Clinical Cancer Centre by the Organization of European Cancer Institutes. The ED is organized in 3 units: Emergency Room, Short-stay Observation Unit, and Emergency Medicine. Staff includes 108 professionals (physicians, nurses and nursing assistants). While medical staff and nursing assistants rotate on all three wards, nurses are permanently assigned to one of them. The ED has approximately 74.000 annual visits. The Clinical Cancer Centre includes a hospital-based PC Unit with no beds, whose mission is to provide clinical consultations for inpatients and outpatients, and carry out training and research activities.

The study protocol was approved by the Ethics Committee of the Provincial Health Authority of Reggio Emilia. Our process was aligned with Standards for Reporting Qualitative Research developed from O’Brien et al. [30]

### Selection of participants

We conveniently selected HPs working at the ED, stratifying them by sex and years of professional experience at the Department. We asked the management of the ED Department for the participation of six physicians, five nursing assistants and 15 nurses (five for each unit). A further sample of potential substitutes was selected, in case of refusal or inability to participate. Participants were invited by e-mail to take part in the study, and non-respondents were then contacted by phone. We also identified a purposive sample of patients and relatives (other than those of patients selected), who had visited the ED within a defined week. Eligible patients were identified by the ED nursing coordinator. Inclusion criteria were: a) patients aged 18 or older, b) with palliative care needs (estimated life expectancy of fewer than 6 months), c) with significant impairment of one or more of the quality of life dimensions, d) having visited the ED and been transferred to another hospital department. The ED nursing coordinator identified also eligible relatives. Inclusion criteria were: aged 18 years or older and had visited the ED with the patient. A researcher contacted eligible patients and relatives by phone during the patient’s hospitalization period, and invited them to participate in the study, providing them with all the information. In case of consent to participate, the researcher made arrangements for a meeting time and place.

### Data collection

The opinions and perceptions of HPs were explored by means of focus groups (FGs), while feedback from patients and relatives was gathered by means of semi-structured interviews. The purpose of the FG was to encourage the interaction between participants to elicit a range of views. Four mono-professional groups were formed, in order to create an open environment that would encourage open and frank communication among the participants. The FG was conducted based on a list of relevant topics to be discussed and included an open question for each issue (Table 1).

**Table 1:** Focus group topic guide

Topic 1 – Critical issues and difficulties	
The facilitator explores the issues perceived by the participants in assisting patients in advanced and terminal stages of illness, visiting the ED. Particular attention is paid to strategies for dealing with the physical, psychological, social and spiritual symptoms of patients and their relatives. Besides, she stimulates the participants to deepen the difficulties through the illustration of actual and lived situations. Prompt: What are the problems associated with assisting patients with PC needs in ED?	
Topic 2 - Reasoning about critical situations	
The facilitator investigates the opinions and the perspectives of the participants about the reasons underlining the difficulties reported. Prompt: What are the reasons behind these problems?	
Topic 3 - Applied coping strategies	
The facilitator explores the cognitive, emotional and behavioural strategies applied to cope with the reported issues. Prompt: How do you face these critical points? What are your experiences? How do you try to solve them?	
Topic 4 - Desired coping strategies	
The facilitator investigates the opinions of the participants regarding other possible ways to cope with the reported issues. Prompt: How do you think these critical issues can be effectively addressed? Which resources/strategies you think are appropriate?	

FGs were held within the ED, at participants’ convenience. Each FG meeting was conducted by a pair of researchers from the Steering Committee of the study (SDL, psychologist, SA, physician, CA, nurse) with expertise in PC and in qualitative research, one with the role of facilitator and the other one as observer. The facilitator introduced the topics, encouraged participation and addressed issues raised by respondents. The observer took detailed field notes on the nature and type of participation of group members, and ensured that discussion was aligned with the aim and the topics of the FG. The day after each FG, the facilitator and the observer met to compile a written report in which salient topics and verbatim responses were set out.

As to the interviews to patients and relatives, these were structured to follow a topic guide (Table 2) to explore the recent experience of the ED visit, with specific focus on problems encountered. Interviewers (CA, MC) were a nurse and a physician with expertise in palliative care and qualitative interviewing. Written informed consent was obtained for all the participants. Explicit permission was given for the interview and the FG to be audio-recorded. Interviewers did not meet and know the participants before the study.

**Table 2:** Patients and relatives’ interview guide

Theme 1: The clinical situation through the words of patients and relatives	
The interviewer explores the problems that motivated the visit to the ED. Sample question: “Could you please tell me about your clinical situation/ the situation of your loved one? Could you please tell me about the visit to the ED?”	
Theme 2: Accessing the ED	
The interviewer investigates on how the patient and relatives feel about the way HPs assessed the patient’s symptoms and the relationship established with the HPs. Sample question: “Could you please tell who welcomed you? What questions did the HP ask you? Have the questions been asked directly to you or your family (if any)? How did you feel in that situation? What about the time dedicated to you/your loved one?”	
Theme 3: The perceived quality of care	
The interviewer explores the patient and relative’s perspective on the quality of the assistance received, by focusing on their perception of how physical symptoms were addressed, on attention HPs gave to psychological and spiritual aspects, and their information needs. Sample questions: “Did the nursing staff meet appropriately the needs? How did they deal with your situation/the situation of your loved one? Did doctors, nurses and other team members take effectively care of the problems? Could you please tell me about the timing? What about the information they gave? Information about the nature of your/relatives’ problems? The therapies or treatments? Did you feel involved in the decisions about your/loved one’s health?”	
Theme 4: Overall evaluation	
The interviewer asks participants to comment on the experience at the ED. Sample questions: “In your opinion, what is ‘quality’ care like? How do you evaluate the quality of care received at the ED?”	

### Data analysis

Both FGs and interviews have been transcribed verbatim. The researchers did not return transcripts to participants. Both datasets were analyzed using thematic analysis [31]. SDL, CA and SA transcribed the recordings verbatim; they then read it with GM several times. Field notes were used to evaluate any insightful non-verbal behavior, setting the focus of the coding process on manifest and latent contents of the transcripts. Each transcript was labeled independently by two researchers, who met to discuss the initial codes. Then, labels were combined to identify the core themes and sub-themes. The two researchers brought their analysis and discussed to reconcile differences in labeling. The themes were further reviewed by LG and GC to ensure accuracy. The list of topics identified was discussed with FDV (psychologist). Afterward, it was reviewed and refined to assure its internal coherence. Finally, the same thematic analysis was applied to both the datasets in order to evidence similarities and differences between providers’ and users’ perception.

## Results

### Characteristics of study subjects

Of the 26 selected professionals, 21 participated in the FG meetings (4 physicians, 12 nurses, 5 nursing assistants). Two professionals who could not participate in the FG meetings, for not-disclosed reasons, were replaced with their substitutes; however, five could not be replaced as they did not provide timely notice for researchers to contact substitutes. The mean duration of each FG was 57 min. Semi-structured interviews were performed with six patients and five relatives, 1 or 2 days after transfer from ED to another hospital ward. Table 3 reports the main characteristics of interviewed subjects. There were not repeated interviews.

**Table 3:** Characteristics of interviewed subjects (*n* = 11)

Characteristics of interviewed subjects	Values
Participant type, N (%)	
Patient	6 (54.5)
Relative	5 (45.5)
Age	
Patients’ average	68 (range: 49–86)
Relatives’ average	63 (range: 47–69)
Gender, n %	
Female	9 (81.8)
Male	2 (18.2)
Patients’ primary disease, n (%)	
Cancer	6 (54.5)
Pulmonary disease	5 (45.5)
Primary reason for visiting the ED, n (%)	
Pain	2 (18.2)
Breathlessness	4 (36.4)
Gastrointestinal problems	3 (27.2)
Ambulation problems	2 (18.2)
Interview duration (mean)	15 min (range: 9–24)

### Main findings

Several common topics were identified and grouped under five core themes: shared priorities in ED among professionals and patients, the information provided by professionals and that desired by relatives, perceptions of environment and time, limitations and barriers to the continuity of care and the contrasting interpretations of giving and receiving PC. The following paragraphs report on insights into themes and sub-themes that emerged, pointing out commonalities and differences between the different ‘actors’ (i.e. providers and users) involved in the experience of caring and being cared for when dealing with an advanced chronic-degenerative illness within the ED. These themes, together with their component sub-themes, are described more in detail in Table 4, where representative quotes from participants were also reported.

**Table 4:** Themes, sub-themes and representative quotations from qualitative analysis

Themes	
Sub-themes	Representative quotations
Shared priorities in ED among professionals and patients	
Patients’ priority is being relieved from their physical suffering	“We don’t see much of the patient when he/she isn’t feeling well” (physician). “If you ease at least some of the pain you can take away, they don’t ask you information...” (physician) “The patient is in pain, so we administer an analgesic, or morphine...” (nursing assistant) “I can’t really tell you they asked me any questions understand the problem, since, I was confused but I heard the nurse talking and asking about my problems. I practically had a foot in the grave … [*omitted*] I can’t explain. I felt I was going to die!” (patient) “They didn’t give me information about my problems, but really I didn’t ask them anything. This is because I understood my trouble even before the doctors.” (patient) “The assistance I received was great; I went in gasping for air and went out breathing!!” (patient)
Being emphatic/ being perceived as emphatic	“We struggle to put up a barrier between us and the patients. We experience death in the emergency room with multiple trauma. I don’t know if it’s just my personal opinion, but I have less issues dealing with a young patient with multiple trauma then with an oncological patient.” (nurse) “[Patients] are people to whom I really don’t know what to say. I think: ‘I really don’t know what to tell you, and actually I just feel better if I didn’t see you!’ Not to mention when they ask questions... like “will I get better?” When will I get better? What happened to me?’ ‘Why don’t you ask my colleague, it’s better...!” (nurse). “Sometimes we shield ourselves behind the fact we’re busy carrying out our work [*omitted*] trying to avoid that aspect, for many reasons; hoping the patient will get transferred as soon as a free bed comes available [*omitted*], so we get rid of the problem, for many reasons, because we’re uncomfortable, we don’t know how to act...”. (nurse) “I felt everything was going well, that I was already under their protection!” (patient) “They have acted kindly and professionally. [*omitted*] Anyway there were four or five doctors around my wife’s bedside, so I guess I should at least be happy because they were following her” (relative)
The information provided by professionals and that desired by relatives	
Relatives’ expectations towards ED professionals	“... And when you hear them say “why aren’t you doing anything? Isn’t there anything left to do? You’ve got to do something! [*omitted*]There is this perception that no medical action is being undertaken to care for the patient who is suffering, because they cannot understand how far medicine can go and where it takes something else” (physician) “When someone goes to the ED, he/she is convinced that a doctor could tell him/her: “You have a problem and I’ll heal you! Unfortunately, this is not always possible. But we, and the patients want to be cared for immediately, and want to be sure of healing. … Unfortunately, this is not possible in life…” (relative)
Why do relatives repeatedly ask for the same information?	“… Questions that make you understand that they really weren’t expecting it, and that they were absolutely unaware of the stage of the disease… or simply they need to ask questions hoping to hear you say that it’s not true, so you really don’t know what to reply...” (nurse) “Many times the fact they say “we don’t have any information”, is not because they don’t have information, rather that they don’t not want to know the whole story, or they don’t want to remember it” (physician) “We didn’t ask anything… [*omitted*] The moment we left home we felt it would be a last trip, so many times we didn’t want to ask to avoid bothering anyone…[*omitted*] “We were already prepared to this moment …so we didn’t need to ask …” (relative)
Waits time before being informed/ receiving information	“You have to repeatedly ask them [the physicians] 1,2, 3, 4 times … “Have you spoken with a relative?’ They are always so caught up in the scientific aspect alone...” (nurse) “[the patient] came in and was really in bad shape, and I saw that we didn’t wait long. When we arrived he was already inside [*omitted*] They told us ‘a doctor come and call you now’, and indeed a doctor. Came soon after” (relative) “In a second moment when [patient] became conscious they explained to us the first types of treatments that they had administered, and that they would have brought him up into the ward [*omitted*]. Not immediately, but as soon as he got things together… (relative)
Perception of environment and time	
Spaces within the ED	“The Emergency Room, per se, is perhaps the least appropriate place... to approach a topic like this in a complete manner. I’ve heard of that maybe the Short-stay Observation Unit offers spaces with better characteristics... at least on the layout they’ve got what are listed as rooms –although you really can’t call them ‘rooms’– but which in fact are dedicated to these types of patients... You’ve got a different, more direct contact with the family member.” (nurse). “You’ve got a little more time to dedicate the patient [*omitted*] and speak to him/her for a couple of minutes...” (nurse) “I believe it’s matter of dignity being able to deal in a certain way with certain people. [*omitted*] if you have a place that is somewhat more intimate, it’s better.” (nursing assistant) “In the Emergency Room, the only privacy patients have is provided by a curtain [*omitted*], while the patient’s suffering and has all the problems of the world –supposing he/she is even conscious enough to understand what’s going on-, in my opinion it’s a situation a human being does not deserve. (nurse) “Even when you have to give bad news. Where is that given? I feel bad for them… in the hallway! If they give me such a news I’d fall on the floor! Where should I put them? Where should I take them? To the relax room, where we go to have a cup of coffee, which in most cases where is likely have someone walk-in laughing...” (nurse) “The family members find themselves at the end of a painful path, [*omitted*] they’re quite exhausted and proven, therefore they need some privacy...” (nursing assistant)
Time devoted by ED professionals to patients and relatives	“In my opinion we don’t give the patient the time he/she deserves [*omitted*] what I mean there’s a risk that… if one puts themselves in the shoes of this type of patient, that patients feel themselves abandoned!” (nurse) “You’re there busy, feeling your patient’s abdomen, interacting with your colleagues, and meanwhile the family comes up to you for information … You’re doing three things at once!” (physician) “They immediately put on the oxygen mask so he immediately received all the care needed!” (relative) “You are welcomed, everyone takes care of you dance by you to do anything they can. And this it is what I believe to be ‘Quality of care’, it’s not just the treatment but also the way you are treated as a person. Because if you just administer medication and then you leave the patient there… They often come there and stay with you, sometimes pampering you, and it really means a lot to me!” (patient)
Availability of medical devices	“Sometimes that happened to go out in the oncology ward to get some medication and not even know what was inside... (nurse) “The bed is uncomfortable. The stretchers are uncomfortable. Patients have this little stretcher... But then if they need to turn around they can’t because it’s painful. And maybe they might even have sores...” (nurse) “Actually [my wife] was on the stretcher from 8 o’clock to 3.30 in the afternoon. And with the problems that she has and back pain and sore legs, she could not bear it any longer …” (relative)
Limitations and barriers to the continuity of care	
Interprofessional communication outside the ED	“Inside the ED you don’t really diagnose, so you don’t even know the underlying information process...” (nurse) “We work h24 well and oncologists much less, and you don’t even know what has been said to the family, or to the patient. Many times the patient comes in, and 10 min later we’re told ‘the patient doesn’t know anything’” (physician) “Sometimes we received a call from the oncologist who tells us ‘there’s a patient coming in from home’, and most of the cases they don’t bring any medical records, and the oncologist doesn’t provide us with any information” (physician) “Many times we say: [the patient] he’s going to be back tomorrow! Because if he’s not part of a network, or if he has not a support that helps him outside, after the acute episode, he’ll return... (physician) “We work by pre-established patterns…But [*omitted*] the patient must be considered as a whole, and not just as a part alone” (nurse) “The problem is that’s the kinds of patients are changing. We have to change our way of working, approaching patients, of setting priorities, but there isn’t anything [in the system] that changes and allows us to do this in a simpler way” (nurse) “…Pathways that in my opinion are not always correct. I mean these patients arrive from home and are in pain… the burden of a lifetime. They arrive to the oncology day hospital and they stay there all day, being promised that they will be found a bed. Then they are taken to the Emergency Department and they spend a whole afternoon waiting for a bed, and then they’re taken to the Short Stay Observation or in the ward... [*omitted*] And patients go through all this to achieve… what? In my opinion these pathways are not always appropriate” (nurse) “And what about patients from facilities with multiple comorbidities? You can’t find a thing on what the facility and the doctor decided for the patient! What are the family members waiting on??” (physician) “They have little knowledge of the person … as soon as they know about your problems… there isn’t much that they can do …” (patient)
Interprofessional communication inside the ED	“The family members come to speak with the doctors... They go there and you really don’t know what they were told [*omitted*] so then not even we can have a contact with the family members. You are afraid to go into the room and tell them something different! [*omitted*] What’s missing is the contact. Maybe even our fault. But what’s missing is the communication between nurses and physicians. This approach is currently lacking” (nurse) “When doctors change shifts and update their colleagues, it’s not that they come over to you and ask you ‘what do you think about patient?’ ‘Is he in pain?’ Isn’t he in pain? (nurse) “They [the doctors] come and tell us what they decided, but there should be more interaction with nurses to do things well.”(nurse) “It’s not even easy for them, because they don’t know the persons…” (relative)
The contrasting interpretations of giving and receiving PC	
Management of pain and other physical symptoms	“it’s like being in a Third World country. Especially for us, as we don’t even have any instructions to carry forth. It kind of activate instinct...” (physician) “with your own personal experience” (physician) “The first issue is the pain, because after a first attempt –which is mostly based on FANS or paracetamol or when we exaggerate tramadol–using more potent and effective medication takes repeated requests to physicians” (nurse) “[the doctors] are afraid... they administer one cc of morphine at a time...” (nurse) “I believe their fear is linked to the lack of competence” (nurse) “In order to set up a CPAP [*omitted*] you need the patient’s collaboration first of all; otherwise you need to sedate the patient. And in this place we don’t have sedation [*omitted*] Instead of sedating them we tie them... [*omitted*] Poor patients, let’s just sedate them!” (nurse) “What can you expect… they’ve administered some painkiller…[*omitted*] They didn’t do things sloppily… because they don’t know the person…” (patient) “I saw that they really put much effort in dealing with this problem… [*omitted*] I did notice that they are there when there is a need …” (relative)
Training needs	“We’re in 4, 5 different physicians, each being used to manage pain treatment in their own way. Perhaps, it would be better if there were a more standardized approach...” (physician) “I feel a bit inadequate... [*omitted*]. I follow my instinct, I mean when I’m in front of the patient I look at the way he/she reacts, I try to avoid saying nonsense.. But I really feel I am not prepared...” (nursing assistant) “We lack the training on the relational aspect more than on the practical tasks. Because after so long we know how to manage the patient. But we don’t know how to manage the relationship, to what extent we can engage with them... And at what point should someone else intervene?” (nursing assistant) “We only have our human nature to support them [*omitted*] it’s the situation where one human being is telling another that the person they love is about to leave us and there is nothing you can do about that [*omitted*] We communicate directly that the person has passed... We don’t prepare them to the event” (physician) “Training on the multidisciplinary aspects of oncological patient, that means enteral nutrition as well since these cases occurs up frequently” (nurse) “They have all been great, they did what they could, but it’s clear that if you have a severe problem they’ll send you to the ward” (patient) “They worked well…nobody can fight against death…” (relative)

#### Theme 1: shared priorities in ED among professionals and patients

All professionals agreed that their priority in treating a PC patient is to relieve the patient’s physical suffering. Communication, conceived as a set of techniques for receiving and delivering appropriate information, as well as being attentive to the patient’s emotions, was only a marginal issue. This emerged both from the FGs and from the patients’ narratives. As stated by one patient with pulmonary disease “The assistance I received was great; I went in gasping for air and went out breathing!”.

As to the emotional and relational aspects of communication, nurses and nursing assistants repeatedly underlined the uneasiness they felt in dealing with the patient suffering from physical symptoms, trying to find the “right” balance between closeness and distance. Some referred to cope with this difficulty either focusing their interventions on the practical care tasks, or avoiding an empathetic and human contact with the patient: “Sometimes we shield ourselves behind the fact we’re busy carrying out our work [*omitted*], hoping the patient will get transferred as soon as a free bed comes available [*omitted*], because we’re uncomfortable, we don’t know how to act...”. Such uneasiness was not perceived by patients, who, on the contrary, felt satisfied about how they were being cared for. As one woman with advanced cancer stated: “I felt everything was going well, that I was already under their protection”.

#### Theme 2: the information provided by professionals and that desired by relatives

Although HPs did not seem to consider communication with patients an issue, they did feel that there were some critical aspects in communicating with the patient’s relatives and in passing information among colleagues in the ED. Physicians claimed they remain bewildered by the patients relatives’ unrealistic expectations for what can be done, lamenting they were often blamed by the relatives for not doing enough for their loved one. “There is this prejudice: no medical action is being undertaken to care for the patient who is suffering, because they cannot understand how far medicine can go and where it takes something else” as one physician said. Another aspect they agreed on was the relatives’ tendency to repeatedly asking them information concerning their sick family member. Some speculated that this was due to relatives’ human need to protect themselves from traumatic news, others to their hope to see that the information they had received were disconfirmed, others that the information they had received by healthcare professionals other than those within the ED was incomplete or incorrect.

Conversely, the relatives in turn did not perceive any issues with the information being given to them. Also when they said they had not been informed about their loved one’s condition, they justified the situation by saying they already knew what to expect and that they did not feel the need for further: “We didn’t ask anything [*omitted*] The moment we left home we felt it would be a last trip”.

As to the communication among staff members, nurses and nursing assistants complained about physicians’ behavior, who do not update or share with them any information on the patient until speaking directly with the patient’ relatives. On some occasions this could occur after hours, putting the nursing staff in the uncomfortable position of not knowing how to manage the patient’s family who was anxiously waiting in the waiting room. This aspect had not been perceived by the patient’s relatives interviewed.

#### Theme 3: perception of environment and time

Another theme that emerged from our analysis was represented by more practical logistical aspects in which assistance is delivered or received in the ED, such as proper care spaces for patients to receive PC and for family members to wait. All professionals rebuked the totally inadequate spaces of the Emergency Medicine and the Emergency Room Unit for communicating bad news to patients and their families. As one nurse explained “In the Emergency Room, the only privacy patients have is provided by a curtain [*omitted*], while the patient’s suffering and has all the problems of the world –supposing he/she is even conscious enough to understand what’s going on-, in my opinion it’s a situation a human being does not deserve”. One relative claimed that the patient was placed on a very uncomfortable stretcher. Both nurses and nursing assistants confirmed the ED not to be organized to properly manage PC patients, lacking the means to offer proper comfort and sometimes even the drugs used in advanced stage cancer.

The lack of adequate spaces was also mentioned in reference to accommodating family members. Because EDs have not been designed to care for dying patients, there are no waiting rooms other than hallways, which sometimes not even provide basic commodities such as chairs or a coffee table, and even less the private space for receiving bad news. This aspect was not an issue for the HPs in the Short-stay Observation Unit, as the staff had in fact recently set up an area to properly host for PC patients and their family. Logistics, however, was not mentioned as an issue by the patients and relatives interviewed. Agreement among all professionals was also observed when speaking about the lack of time to properly address the special needs of a patient in PC, as well as time to interact with the patient’s relatives especially upon receiving bad news. Physicians described a state of distress as they were constantly engaged in multiple activities at the same time: “You’re there busy, feeling your patient’s abdomen, interacting with your colleagues, and meanwhile the family comes up to you for information”. Yet, on speaking about time, nurses referred another aspect, that is the patient’s waiting time at the ED before being admitted to care. As the nurses pointed out, triage procedures did not keep into due consideration the medical conditions of PC patients, and advocated that priority codes be revised accordingly.

Again, time constraints or waiting times did not represent an issue for the patients and family members, rather they highlighted the “quality” time they received at the ED: “They often come there and stay with you, sometimes pampering you and it really means a lot to me”. Only one family member lamented that his relative had to wait an extremely long time before being moved to another ward.

#### Theme 4: limitations and barriers to the continuity of care

Continuity of care to the patient presenting at the ED was a critical aspect. One of the most evident problems was the lack of disease-related information from the oncologist or their general practitioner (GP) on the patient’s digital clinical records. As physicians stated, the oncologists often failed to insert information on ongoing pharmacological treatments, stage of disease and other relevant information that was necessary to other HPs who might have to deal with the patient outside the oncology ward. The sharing of information hence often relied on phone contact, however many physicians were difficult to reach at out of office hours, especially on holidays. Accordingly, this lack of access to updated information prevented the ED staff to offer continuity of care to the patient. This issue was a main critical point, especially as high numbers of severely sick patients with multiple health conditions presented repeatedly to the ED. Continuity of care as to palliative treatments was only one side of care; the other was the ethical aspect of the patient’s will and end-of-life care. As stated by one physician: “And what about patients from hospices with multiple comorbidities? You can’t find a thing on what the hospice and the doctor decided for the patient!”. Both patients and relatives seemed to perceive the poor knowledge of patient’s clinical condition by professionals as a sort of intrinsic limit of the assistance within the ED. Continuity of care is felt by nurses and nursing assistants to be compromised not only for difficulties in inter-professional communication outside the ED, but also by communication issues within. They reported on the lack of contact and dialogue with medical staff, particularly with reference to the poor consideration of their assessment of patients’ pain: “They [the doctors] come tell us what they decided, but there should be more interaction with nurses to do things well.” reported a nurse. Inter-professional communication outside and inside the ED was not perceived by patients.

#### Theme 5: the contrasting interpretations of giving and receiving PC

Physicians expressed their awareness of having a poor knowledge on pain management of patients with incurable illness. This was also stressed by nurses, who further highlighted the physicians’ lack of competence in the proper management of other physical symptoms in patients with PC needs, such as constipation and restlessness. All professionals stressed the need to receive specific training on therapies and medical devices aimed at optimizing pain management in advanced cancer patients, also through the involvement of the hospital Palliative Care Unit not only for clinical, but also for educational purposes. Patients in turn reported on the benefits they had relief from the symptoms causing them severe suffering, and from fear of death. Moreover, all patients and most relatives highlighted that they were satisfied with the assistance and care received within the ED, and satisfaction was expressed even when physical symptoms were not completely managed by ED staff: “They worked well…nobody can fight against death…” explained a relative.

Physicians strongly emphasized their lack of skills in delivering bad news and in dealing with the psychological impact of such news on patients and relatives, “We only have our human nature to support them [*omitted*] it’s the situation where one human being is telling another that the person they love is about to leave us and there is nothing you can do about that a physician told us”. Both nurses and nursing assistants advocated the need for training to overcome communication problems within the ED, suggesting a continuous and repeated training over time, including practice sessions. The implementation of framework similar to training received on the clinical management of patients with acute injuries emerged as desirable by participants.

## Discussion

As reported by many studies, providing PC within EDs may be challenging [32–35]. Although models have been suggested for this purpose [17], their implementation may be difficult due to the following contextual factors: lack of the ED’s access to relevant patient information [36], difficult conversations about goals of care among physicians [20], patient and family emotional distress [37], and a number of environmental issues such as noise, time limitations and lack of privacy [23, 38]. Such aspects were also confirmed by our study, and evidenced three main areas within the Italian ED context that need to be addressed and improved, i.e. a) the training for ED professionals on PC, b) the development of a shared care pathway for patients with PC needs, and c) the optimization of ED environment.

With reference to the first area, both patients and HPs conceived the relief from physical symptoms as higher in their priorities than communication issues. Physicians admitted they felt their skills in pain management were inadequate; moreover, both nurses and nursing assistants reported they felt physicians had a reluctant behavior and scarce competency in caring for patients’ physical suffering. Surprisingly, and contrary to the results from other studies [4, 14, 26, 27], all patients and most relatives were satisfied with assistance received within the ED, also where the patients’ physical symptoms were not completely relieved. In line with a quality improvement approach, focused on the appropriateness of the care provided rather than on users’ satisfaction, our results emphasize the importance of making ED staff competent and confident in appropriately managing the problems of these patients and their relatives. This could be achieved through training programs specifically developed for ED professionals, a priority claimed in several studies as a compelling requirement for them [15, 16, 21]. According to HPs’ views in our study, such training should be continuous and include not only theoretical but also experiential modules on the prompt management of physical symptoms, as well as on difficult communication scenarios, particularly with relatives.

With reference to the second area, the lack of a pathway was justified or expected as intrinsic limitation within the ED by patients participating in our study, which again is in line with literature documenting that patients’ perceptions of continuity of care varies according to both individual and contextual factors [39]. HPs had a different perception: the lacking of a pathway as a barrier to the delivery of appropriate care, denouncing both the unavailability of clinical documentation concerning patients visiting the ED and the difficulty in tracking the patient’s oncologist or GP to obtain information on the patient. These issues were also documented in other studies reporting the opinions of HPs caring for people with advanced cancer and older people presenting to EDs [16, 40], where collaborative processes, constant communication among different professionals and defined pathways were identified as priorities for improving ED-based PC. Thus, the need for the development and implementation of a shared care pathway for patients with advanced illness could have a positive influence on ED in terms of quality of the care provided to such patients and their relatives.

Concerning the third area, as emerging from the FGs environmental aspects represented a relevant issue in clinical practice, as it prevented HPs from carrying out their job properly and contributed to their feeling of frustration and inadequacy towards the patient. Our findings evidenced that the apparently trivial aspect – the lack of physical and emotional spaces – actually compared to not having a basic working instrument available. Despite this opens to the broader issue of architectural planning of places of care in general, the observations raised by HPs reconnect and are intrinsic to the purpose and mission of PC, as by its current definition [1]. Considerations on the impact of environmental factors on the quality of care emerging from our study were consistent with literature, reporting either on the lack of private spaces providing confidentiality, and time pressure made on staff, and the long waiting times claimed by patients [15, 16, 21, 22, 24]. Thus, the environment should be taken into account to implement effective quality improvement projects and to advance ED as a place capable to embrace both staff’s comfort and emotional aspects of patients with PC needs.

### Limitations

Study participants were recruited at a single center, acknowledged as a research hospital in the discipline of oncology and equipped with a Palliative Care Unit. This may affect the generalizability of our findings. FGs were attended by fewer participants than expected; nevertheless, we gathered a considerable amount of information from different professionals working within all three ED units.

Our study was performed on patients and relatives of patients having moved from ED to another hospital department. We did not know if their feedback could be different in case of discharge.

Most interviews were performed by a single researcher (CA), a palliative care nurse with expertise in qualitative methodology. Nevertheless each interview, as well as each FG, was analyzed by couples of researchers, and the analyses discussed together with the whole research group, ensuring the rigor of the research process. Both patients and relatives responded in a concise way to prompts and questions of the interviews, and seemed to show some difficulties in focusing the time of stay in the ED (recall bias). This inevitably made the amount of data deriving from interviews less rich than that deriving from FGs. Still, researchers did not force the participants, respectfully listening to what spontaneously emerged from their stories. Moreover, despite conciseness, participants clearly expressed their views as well their perceptions.

## Conclusions

The present qualitative study reveals how PC is acknowledged in the ED, but still far from a full and addressable integration. Indeed, ED physicians, nurses, nursing assistants, patients and their relatives reported several and different challenges, missing points, and criticalities. These reflect targets for improvements, which may help researchers and practitioners to fill the gap and to deliver high-quality PC in the ED. Findings of this study, together with those from literature review on existing interventions to improve PC in EDs, not yet published, will be the basis for the development of a quality improvement program in Italian EDs according to the MRC Framework for the development of complex interventions.

## Data Availability

We do not have ethics approval to make raw data from this study available for sharing.

## Abbreviations

ED: Emergency Department
FG: Focus group
HP: Healthcare professional
PC: Palliative Care
